# 

**Published:** 1879-11

**Authors:** 


*CONTENTS.*

| | PAGE |
|---|---|
| THE EARLIEST SIGN OF CONSUMPTION | 1 |
| SELF-DESTROYERS | 3 |
| MILK SICKNESS | 6 |
| LEAD PIPE | 7 |
| HOMER | 7 |
| PESTILENCE AND PLAGUE | 9 |
| HEALTH AND DISEASE | 9 |
| COLDS PREVENTED | 10 |
| MENTAL REST | 12 |
| BRAIN AND BODY WORK | 14 |
| THE GERM THEORY | 17 |
| CHRISTMAN | 20 |
| HEALTH AND HOUSE HUNTING | 23 |
| FLOWERS | 27 |
| CLOCKS MADE OF FLOWERS | 28 |
| COLD PHILOSOPHY | 29 |
| SUMMER COMPLAINT | 31 |
| SICKNESS AND DEATH | 32 |
| BRAVERY | 32 |

